# Complexity and plasticity in honey bee phototactic behaviour

**DOI:** 10.1038/s41598-020-64782-y

**Published:** 2020-05-12

**Authors:** Morgane Nouvian, C. Giovanni Galizia

**Affiliations:** 1 Department of Biology, University of Konstanz, Konstanz, Germany; 2 Centre for the Advanced Study of Collective Behaviour, University of Konstanz, 78464 Konstanz, Germany

**Keywords:** Visual system, Classical conditioning, Behavioural ecology

## Abstract

The ability to move towards or away from a light source, namely phototaxis, is essential for a number of species to find the right environmental niche and may have driven the appearance of simple visual systems. In this study we ask if the later evolution of more complex visual systems was accompanied by a sophistication of phototactic behaviour. The honey bee is an ideal model organism to tackle this question, as it has an elaborate visual system, demonstrates exquisite abilities for visual learning and performs phototaxis. Our data suggest that in this insect, phototaxis has wavelength specific properties and is a highly dynamical response including multiple decision steps. In addition, we show that previous experience with a light (through exposure or classical aversive conditioning) modulates the phototactic response. This plasticity is dependent on the wavelength used, with blue being more labile than green or ultraviolet. Wavelength, intensity and past experience are integrated into an overall valence for each light that determines phototactic behaviour in honey bees. Thus, our results support the idea that complex visual systems allow sophisticated phototaxis. Future studies could take advantage of these findings to better understand the neuronal circuits underlying this processing of the visual information.

## Introduction

Phototaxis is the movement of an organism in response to light, whether it goes towards it (positive phototaxis) or away from it (negative phototaxis). It is widespread in the animal kingdom and typically understood as an innate, stereotyped, relatively simple response to light. Nonetheless, this behaviour can be modified. For example, whether light attracts or repels an animal can change during its lifetime, especially if the animal occupies different environments as it ages: some insects switch from positive to negative phototaxis towards the end of their larval stage, as they migrate to a dark refuge (e.g. the soil) to pupate^1,2^. Young nurse bees performing indoor tasks are negatively phototactic, while older foragers are attracted to light^3^. At smaller time scales, phototactic behaviour may follow a circadian rhythm, as shown in larval fruit flies^4^. Adult *Drosophila* also shift their preference depending on their internal state: they are normally attracted to light, but will avoid it if their ability to fly is compromised^5^.

Phototaxis only requires very simple eyes^6^, but it did not disappear with the evolution of complex, image-forming eyes such as the honey bees’. There, the architecture of the visual system could potentially support more elaborate forms of phototaxis, especially when multiple photoreceptors responding to different wavelengths are involved. In frogs, grasshoppers, psyllids, *Daphnia*, and *Drosophila*, preference for a particular wavelength can override the intensity signal, in that a specific wavelength can be chosen over a more intense stimulus, either broadband or of another wavelength^7–10^. Even more strikingly, different wavelengths can elicit opposite responses: larval zebrafish are attracted to wavelengths above 400 nm, but avoid those below^11^. The whitefly *Trialewodes vaporariorum* similarly avoids a chamber illuminated by a 400 nm light, but enters one illuminated with a 550 nm light^12^. Mixing the different wavelengths results in an intermediate response in these two systems, suggesting that the lights activated competing mechanisms to produce the output behaviour. Polarization information may also influence phototaxis^8^.

**Figure 1:**
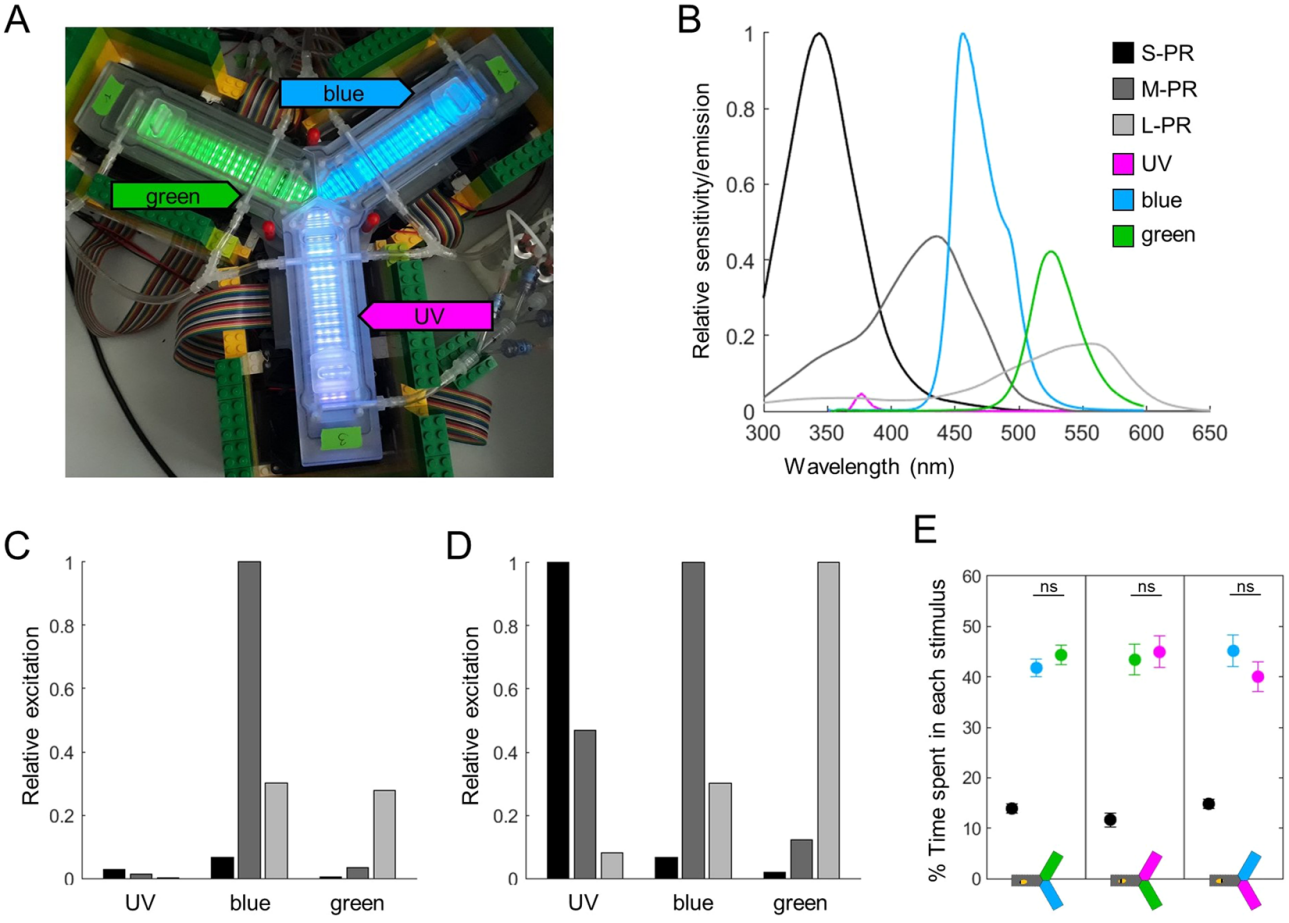
Light stimuli. (**A**) The training and testing apparatus, with the different lights on: green (520 nm) in arm 1, blue (470 nm) in arm 2, UV (375 nm) in arm 3 (the intensities do not match what was used for experiments). (**B**) Relative sensitivity of photoreceptors (after Neumeyer *et al*., 1980) and relative emission of the different LEDs used. (**C**) Relative excitation of the 3 photoreceptors by each type of LED, normalized across stimuli. (**D**) Relative excitation of the 3 photoreceptors by each type of LED, normalized for each stimulus independently. (**E**) Lights were calibrated so that bees spent an equal amount of time in any two wavelengths during the tests (n = 18 bees for each wavelength pair, each bee was tested 16 times with the same wavelength pair). Wilcoxon signed rank tests corrected with FDR, ns: p > 0.05.

The visual system of the honey bee has been extensively studied^13,14^. Bees have three types of photoreceptors: the short-wavelength-receptor (S-receptor) peaks in the UV (344 nm); the middle-wavelength-receptor (M-receptor) peaks in the human blue (436 nm) and the long-wavelength-receptor (L-receptor) is maximally sensitive in the human green (544 nm; Fig. 1B)^15,16^. All three photoreceptors contribute to phototaxis^17,18^, hence this behaviour can be elicited by wavelengths throughout the full visual spectrum of the honey bee. We postulate that this elaborate visual system can support finely tuned phototactic behaviour. Fine tuning could be predetermined, such as wavelength specific behaviours linked to the ecological context in which each wavelength is predominantly encountered. On top of this, individual experience could be integrated to further adapt phototactic responses to the current environment. Indeed, honey bees are well-known for being able to perform a variety of visual learning tasks: they can recognize patterns, shapes, colours and even concepts^19–22^.

The aim of this study was to determine if these two properties, namely wavelength-specificity and plasticity, could be found in honey bee phototactic behaviour. To tackle this question, we performed a systematic study in an automated y-maze (Fig. 1A) equipped with 3 types of LEDs (UV, blue and green) predominantly activating each of the three photoreceptor types (Fig. 1C,D). Using these three light stimuli validated the first part of our hypothesis: differences between the behavioural responses to each wavelength could be readily measured. In addition, we explored how individual experience reshaped (or not) phototactic behaviour. In this instance, we investigated experience-dependent tuning by looking at the modification of phototaxis after simple exposure or aversive conditioning (happening within several minutes), but also by characterizing shorter-term modifications visible within the tests duration (20 s). We found that honey bees can indeed adapt their phototactic responses depending on their previous experience with a particular wavelength, and that this modulation is again wavelength-specific. Finally, we show that other information such as light intensity is also integrated into an overall valence for each light. Thus, our results support the notion that complex visual systems allow for phototactic responses that go far beyond a simple reflex behaviour.

## Results

### Absolute phototactic behaviour depends on wavelength and past experience

**Figure 2:**
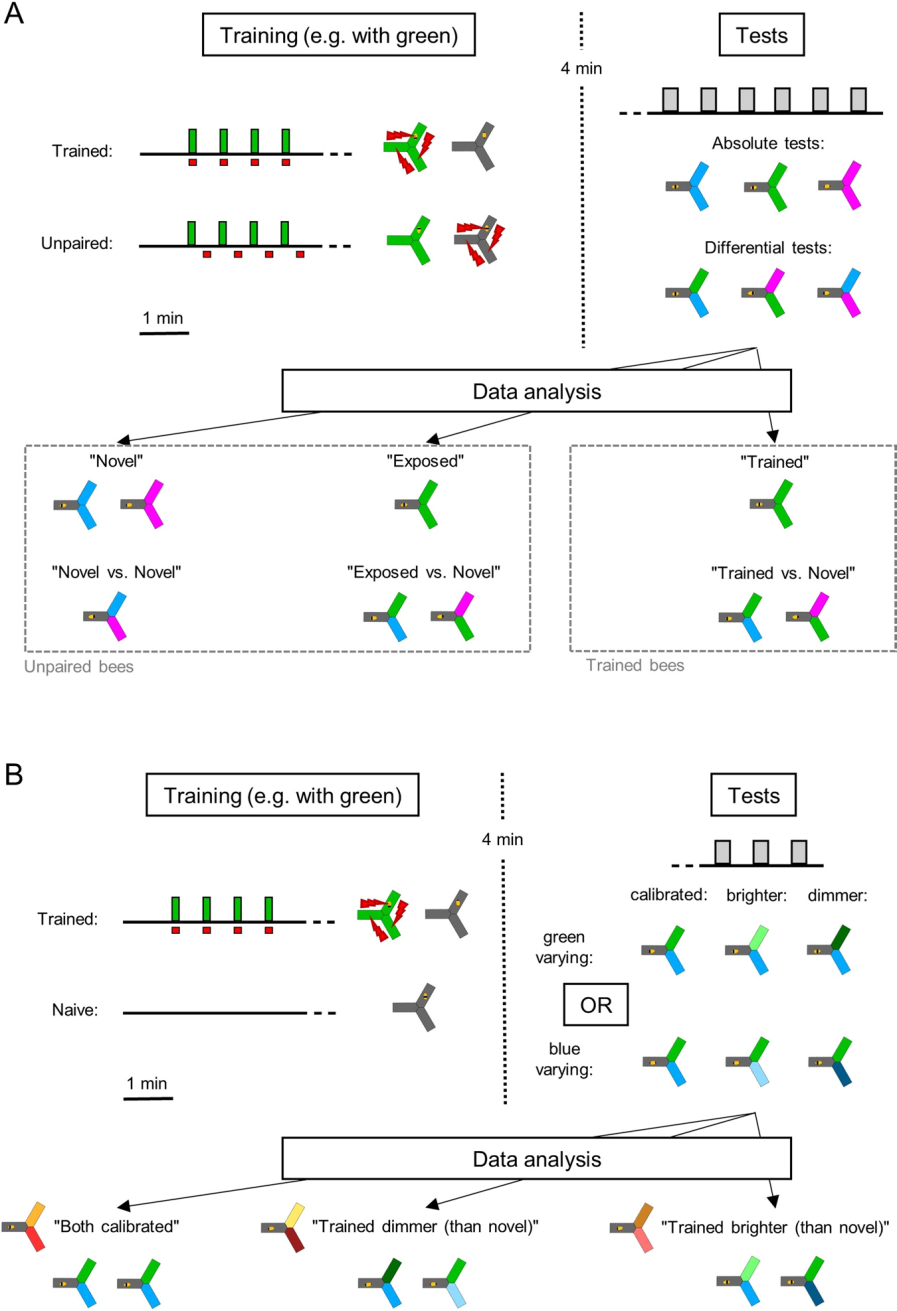
Experimental design. (**A**) Main experiment. First, all bees had 1 min to habituate to the apparatus in the dark. In the trained group, a wavelength (green in this example) was then paired with electric shocks (4 × 10 s, spaced by 30 s in the dark). The unpaired controls also experienced both the light and the shocks, but these were delivered in the middle of the 30 s inter-trial interval. After a resting period of 4 min in the dark, all bees were tested 6 times for their wavelength-preference and phototactic behaviour (20 s tests separated by 30 s). During each test, the bee always started in the dark arm and could choose to enter two lit arms, which could be of the same wavelength (absolute tests) or of different wavelengths (differential tests). For subsequent data analysis, we differentiated the behaviour of the bees towards novel wavelengths, towards a wavelength they had seen but was not paired with shocks (exposed) and towards the wavelength they had been trained to. A total of 6 groups (trained + unpaired for all 3 wavelengths) of 96 bees each participated in the experiment. B. Experiment with varying light intensities. During the first phase, trained bees received shocks paired with one wavelength as before (green in this example). Naive bees spent the same time inside the apparatus but were not stimulated. All bees then participated in 3 tests, during which only one of the wavelengths varied in intensity. For analysis, the data was pooled such that “Trained dimmer (than novel)” included both “bright trained vs calibrated novel” and “calibrated trained vs dim novel” configurations (and vice-versa for “Trained brighter (than novel)”). Half of the naive bees (randomly selected) were attributed green as “trained” wavelength, the other half blue. Since there were 3 different trainings possible (naive + green trained + blue trained) and 2 wavelengths varying (blue varying + green varying), a total of 6 groups of 48 bees each participated in the experiment.

In its simplest form, phototaxis consists in making a choice between light and no light – what we called “absolute phototaxis”. Does absolute phototaxis depend on wavelength? And can it be modulated by exposure or aversive training against a specific wavelength? To answer these questions, we trained bees in the yAPIS (Fig. 1A)^23^ using a classical aversive conditioning design (Fig. 2A): we paired electric shocks with a conditioned stimulus (CS, here a light) for trained bees, while control bees received both shocks and CS but not in close temporal association (unpaired group). We then analyzed their behaviour during tests in which the choice arms were lit with the same stimulus. Depending on the training protocol, this stimulus could thus be: 1) novel (N) to the bees, meaning they had not seen this wavelength before (but they had seen a different wavelength, and received unpaired electric shocks), 2) they were exposed (E) to this particular wavelength and to shocks but not in close temporal association, or 3) they were trained (T) by pairing this wavelength to electric shocks (see Fig. 2A for an overview of the different groups). Importantly bees never received shocks during the tests, so they had no incentive to move towards the lights other than phototactic drive.

**Figure 3:**
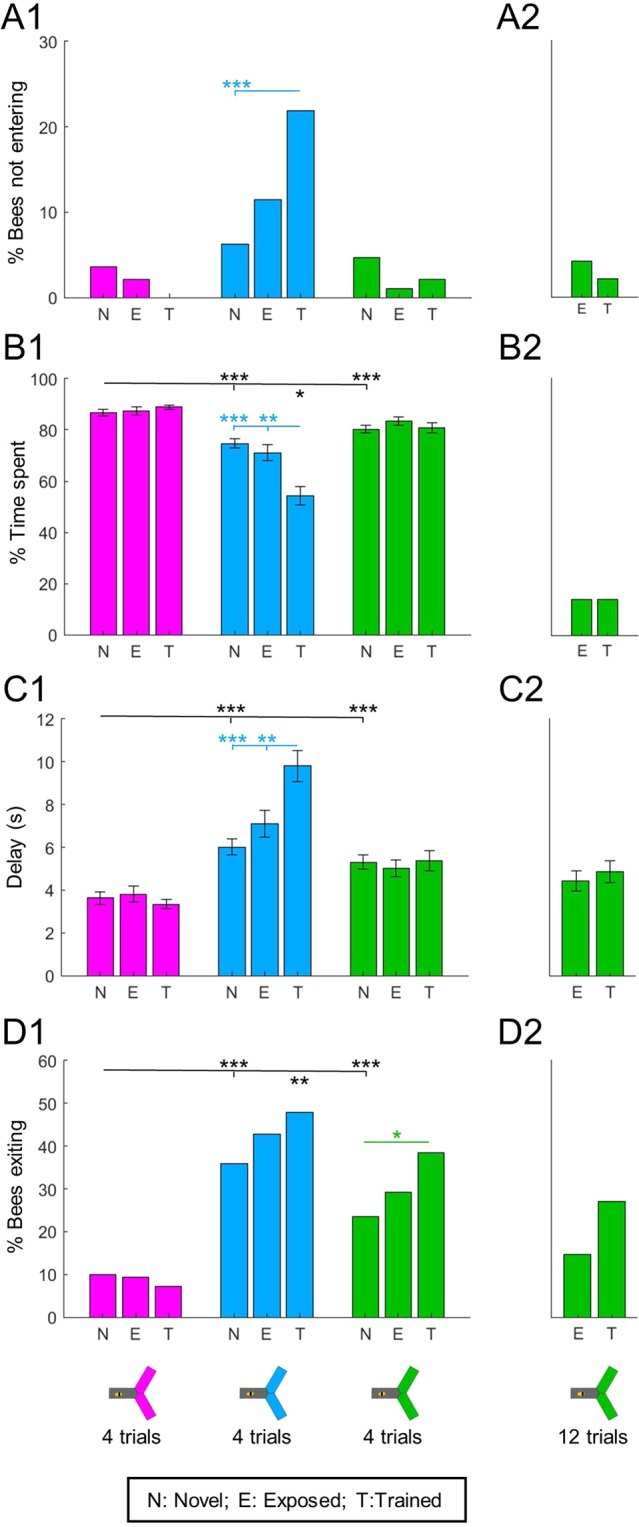
Absolute phototaxis depends on wavelength and previous experience. Absolute phototactic behaviour was tested for all 3 wavelengths and for all possible experiences with each wavelength: novel (N), exposed (E) or trained (T) – from unpaired or trained bees, see Fig. 2A. The main experiment is presented on panels A1-D1. It was repeated with more exposure/training trials for green (12 instead of 4): this 2^nd^ dataset is presented in panels A2-D2. (**A**) Less than 5% of bees did not enter novel lights. The proportion of bees refusing to enter only increased after training with blue. χ^2^ tests. (**B**) The percentage of time spent in the lit arms was different for each wavelength. For the blue light only, training decreased the time spent in the lit area. Wilcoxon signed rank tests, corrected with FDR. (**C**) Delay before entering the lit arms after light onset. Bees entered the UV light faster than blue or green. An increased delay after aversive training was only observed for the blue light. ANOVA followed by pairwise Tukey’s HSD. (**D**) Percentage of bees that exited the light at some point during the test. The proportion of bees exiting was different for each wavelength. This number increased after training for the green light, and a similar but non-significant trend was observed after training with blue. χ^2^ tests. n_N_ = 192; n_E_ = n_T_ = 96; n_total_ = 288, *p < 0.05, **p < 0.01, ***p < 0.001.

We used light intensities calibrated for equal preference between wavelengths (see Fig. 1E and Methods). Nonetheless, we first checked for wavelength-specificity by focusing on the bees’ behaviour towards novel lights (Fig. 3A1-D1, statistics in black). To evaluate the bees’ attraction to a light, we measured the percentage of bees entering (or not) and the delay to enter as proxys for initial attraction, and the percentage of time spent into a light and of bees exiting the light as proxys for how this attraction persists over time. All lights were clearly attractive as nearly all bees entered them (Fig. 3A1), but bees spent longer in the UV than in the blue or green light, and longer in green than in blue (Fig. 3B1; Wilcoxon signed rank tests corrected with FDR, UV vs blue: z = −7.054, p < 0.001; UV vs green: z = −5.671, p < 0.001, blue vs green: z = −2.394, p = 0.017). This could in part be explained by a shorter delay in entering UV than blue or green (Fig. 3C1; ANOVA, F(2,573) = 13.99, p < 0.001, followed by pairwise Tukey’s HSD, UV vs blue: p < 0.001; UV vs green: p < 0.001, blue vs green: p = 0.282). Furthermore, about a third of the bees (36%) left the blue arms before the end of the test, against less than 10% for UV and 23% for green (Fig. 3D1; Cochran Q test Q(2,192) = 48.32, p < 0.001, post-hoc χ^2^ corrected with FDR, UV vs blue: χ^2^ (1,192) = 36.855, p < 0.001, UV vs green: χ^2^ (1,192) = 12.675, p < 0.001, blue vs green: χ^2^ (1,192) = 7.186, p = 0.073). A clear overall pattern emerges from this data: when the stimulus is novel, UV has the strongest phototactic strength whereas blue has the weakest, with green showing intermediate attractiveness.

Did past experience (exposure or training) affect absolute phototaxis? We found that it did, but again in a wavelength-specific manner (Fig. 3A1-D1, statistics in colour). Attraction to UV was unaffected by past experience, with no significant change detected in any of the 4 measures. However, training decreased attraction to blue, as evidenced by some bees completely refusing to enter this light (Fig. 3A1, χ^2^ corrected with FDR, novel vs exposed: χ^2^ (1,288) = 2.363, p = 0.124, novel vs trained: χ^2^ (1,288) = 15.401, p < 0.001, exposed vs trained: χ^2^ (1,192) = 3.75, p = 0.079). Training also reduced the percentage of time spent in blue (Fig. 3B1; Wilcoxon signed rank tests corrected with FDR, novel vs trained: z = 4.385, p < 0.001, exposed vs trained: z = 3.256, p = 0.002, novel vs exposed: z = 0.313, p = 0.754), and the delay to enter was increased (Fig. 3C1; ANOVA, F(2,381) = 13.13, p < 0.001, followed by pairwise Tukey’s HSD, novel vs trained: p < 0.001, exposed vs trained: p = 0.004, novel vs exposed: p = 0.311). The trend for more bees to exit from the blue light after exposure or training was not statistically significant (Fig. 3D1; χ^2^ corrected with FDR, novel vs exposed: χ^2^ (1,288) = 1.243, p = 0.265, novel vs trained: χ^2^ (1,288) = 3.829, p = 0.151, exposed vs trained: χ^2^ (1,192) = 0.525, p = 0.703). Attraction to green was only marginally affected by training, the only significant change being that more bees made excursions out of this light (Fig. 3D1; χ^2^ corrected with FDR, novel vs exposed: χ^2^ (1,288) = 1.110, p = 0.292, novel vs trained: χ^2^ (1,288) = 7.169, p = 0.022, exposed vs trained: χ^2^ (1,192) = 1.884, p = 0.255). This could have been an intermediate state towards the more pronounced changes observed after training with blue. To test this hypothesis, we repeated the experiment with 2 additional groups of bees (1 unpaired control + 1 trained), but this time the bees received 12 training trials instead of 4 (Fig. 3A2-D2). Even with this increased training, there was only a trend (χ^2^ (1,96) = 2.274, p = 0.132) for more bees to exit the light (Fig. 3D2), the other measurements did not change to a relevant degree. Thus the differences we observed between wavelengths likely represented intrinsic properties of aversive conditioning with each particular wavelength.

### Differential phototaxis can be temporally complex

**Figure 4:**
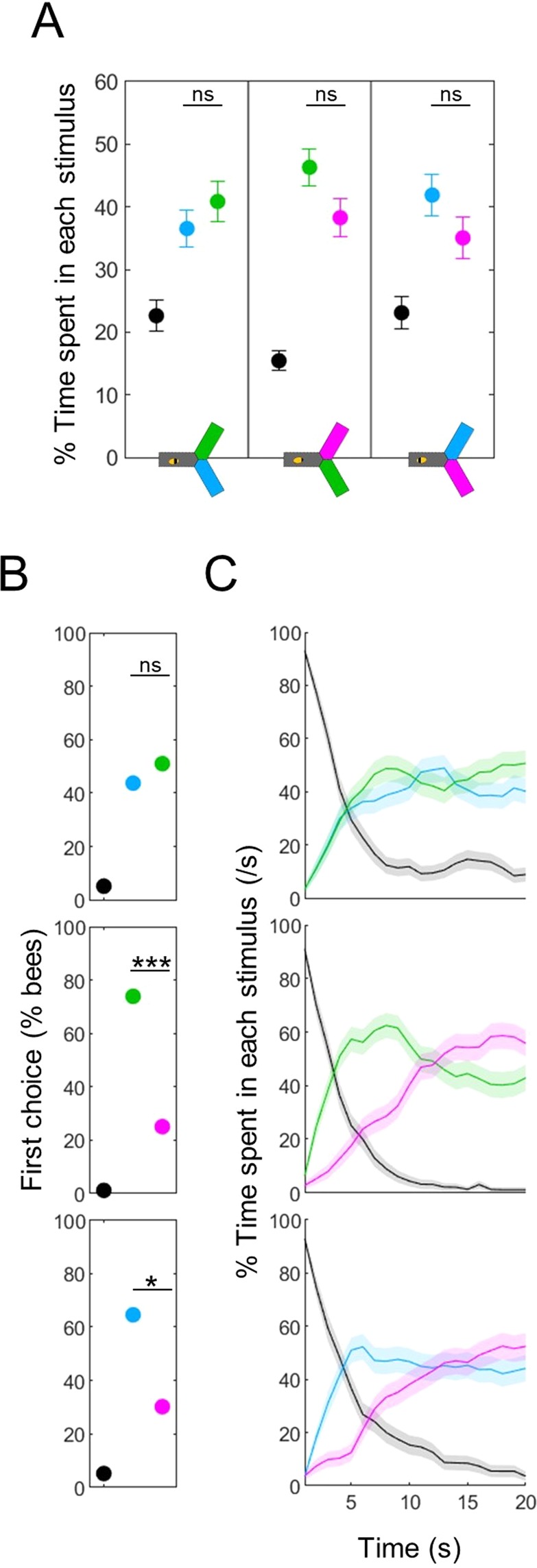
Differential phototaxis has a temporal complexity. (**A**) Percentage of time spent in each stimulus. Control (unpaired) bees presented with a choice between two novel lights spent a similar amount of time in both wavelengths, consistent with the calibration data. Wilcoxon signed rank tests, corrected with FDR. (**B**) Percentage of bees first entering into each light (black = no choice made). The first choice was biased in the tests including UV, such that bees initially preferred the other wavelength. χ^2^ tests against random choices between the two wavelengths. (**C**) Density of bees within each stimulus over time. When the test included UV, bees initially chose the other wavelength but then switched their preference. n = 288, *p < 0.05, ***p < 0.001, ns: p > 0.05.

Even after aversive training, the vast majority of bees still entered the lights during absolute tests (except for blue), such that only quantitative differences could be measured. We reasoned that the phototactic drive may be very strong, and that providing the bees with a second, alternative wavelength could be a more powerful way to uncover a learned aversion (differential phototaxis). Since our analysis of absolute tests hinted at wavelength-specific differences in response delay and persistence, we first looked at the temporal dynamics of differential tests in the cases where the two wavelengths were novel. First, we confirmed that the chosen stimulus intensities yielded equal preference when averaged over 20 s observation time, as per our calibration data (Fig. 4A, Wilcoxon signed rank tests, blue vs green: z = −0.748, p = 0.454, green vs UV: z = 1.310, p = 0.190, blue vs UV: z = 1.171, p = 0.242). For the blue vs green test, the first choice of bees was random (Fig. 4B, top panel, χ ^2^ tests against no bias, χ ^2^(1,96) = 0.27, p = 0.604) and this balance was maintained during the whole test (Fig. 4C, top panel). However, when UV was involved, the picture was quite different: most bees first chose blue or green (χ^2^ tests against no bias, blue vs UV: χ^2^(1,96) = 6.19, p = 0.013, green vs UV: χ ^2^(1,96) = 12.38, p < 0.001). The preference for UV then increased over time, becoming stronger than for the alternative wavelength after 10–12 s. Thus, the test averaged preference hid a complex temporal pattern. It is intriguing that the UV light, which elicited the shortest response delay in absolute phototaxis tests, had slower dynamics in differential phototaxis tests (despite being presented at the same intensity).

### Previously experienced stimuli are less attractive than novel ones

**Figure 5:**
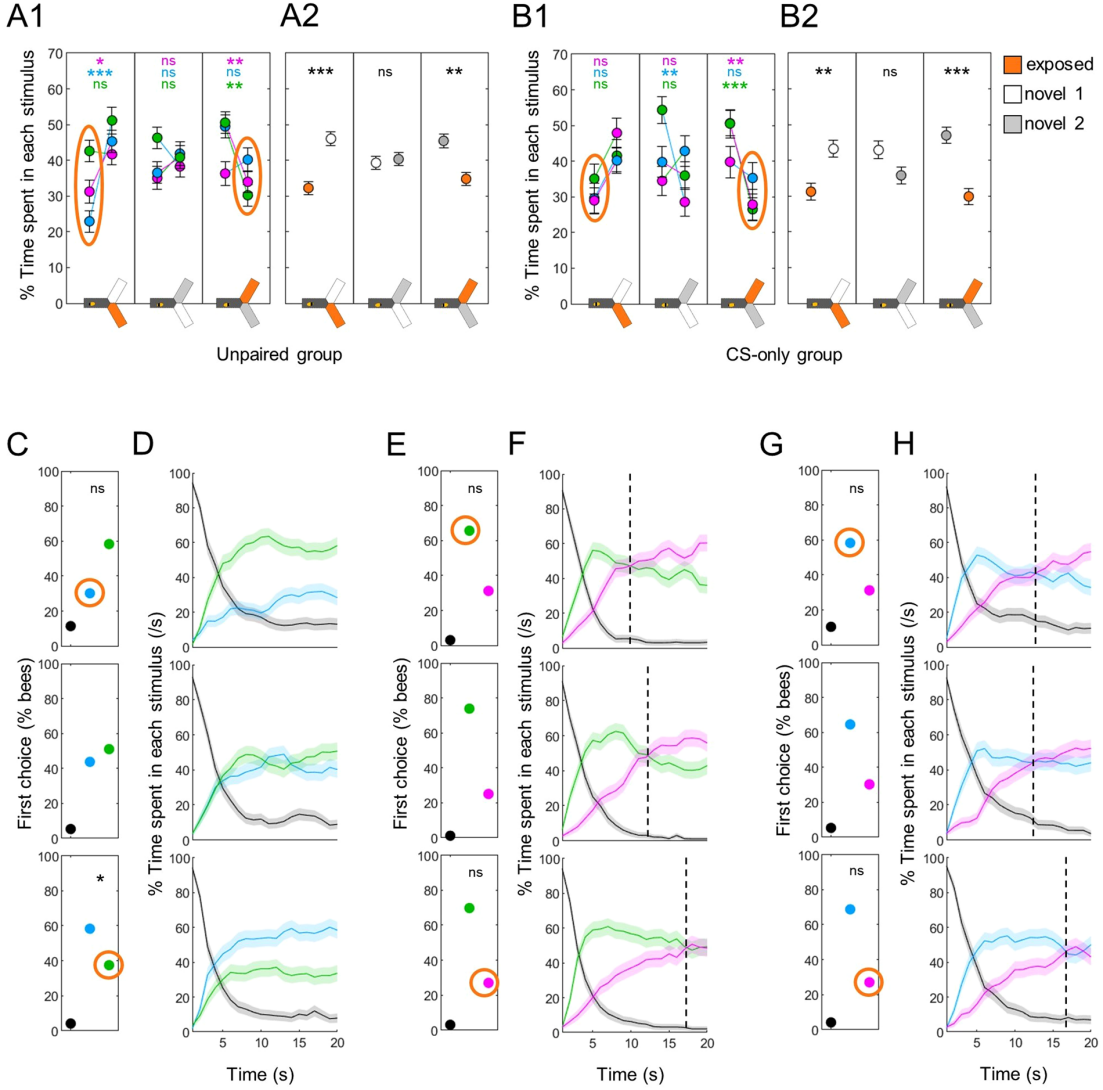
Differential phototaxis is modified by previous exposure. (**A**) Percentage of time spent in each stimulus. A1 presents the data for each individual group (UV-, blue- and green-exposed bees), A2 presents the pooled data. Overall, bees in the unpaired group avoided the wavelength they had seen before. Wilcoxon signed rank tests with FDR correction. (**B**) Same as in A for the CS-only group, in which the shocks were completely removed. A similar avoidance of the seen wavelength was observed. (**C**) First choice in blue versus green tests was also modified depending on experience (seen wavelength circled in orange). χ^2^ tests against bees for which both wavelengths were novel (middle row). (**D**) Time course of blue vs green tests. The bias in preference was maintained throughout the test. (**E,G**) First choices for UV versus green/blue were not modified by exposure. χ^2^ tests against bees for which both wavelengths were novel (middle row). (**F,H**) Time courses of UV vs green/blue tests, respectively. The time at which the switch in preference occurred (dotted lines) was modified by previous exposure to one of the test wavelength. n = 144 for panel B (CS-only groups), n = 288 for all other panels (unpaired groups), *p < 0.05, **p < 0.01, ***p < 0.001, ns: p > 0.05.

Having characterized the baseline behaviour of bees during differential tests allowed us to investigate how it was modified by previous experience with a wavelength. Our first question was: can simple exposure to a light modify phototaxis? To answer this question, we analyzed the responses of bees that experienced both one wavelength and the shocks, but separated by a delay of 10 s to prevent association between the two events^24^. These bees spent less time in the exposed wavelength than when both were novel, irrespective of the wavelength (Fig. 5A1). This was easier to see when pooling the data (Fig. 5A2, Wilcoxon signed rank tests corrected with FDR, exposed vs novel 1: z = −3.880, p < 0.001, exposed vs novel 2: z = 2.960, p = 0.005, novel 1 vs novel 2: z = −0.303, p = 0.762). Could it be that 10 s spacing was not enough to prevent an associative memory? To check this, we repeated the experiment but removed the shocks completely (CS-only groups, n = 48 bees for each wavelength). Again, bees preferred a novel wavelength over the one they had been exposed to (Fig. 5B1–2, Wilcoxon signed rank tests corrected with FDR for pooled data, exposed vs novel 1: z = −2.811, p = 0.007, exposed vs novel 2: z = 3.890, p < 0.001, novel 1 vs novel 2: z = 1.580, p = 0.114). Thus, visually experiencing a light was sufficient to reduce the attractiveness of that particular wavelength.

This bias in preference was also visible in the test dynamics. For differential phototaxis between blue and green, the previously exposed stimulus was already less preferred as first choice (Fig. 5C, χ^2^ tests against bees for which these stimuli were both novel, blue seen: χ^2^(1,192) = 2.64, p = 0.104, green seen: χ^2^(1,192) = 3.98, p = 0.046, the reference bees are plotted again in the middle row), and this remained throughout the 20 s observation time (Fig. 5D). When UV was involved the qualitative effect was different: the first choice remained biased toward the alternative stimulus, with no statistically significant shift (Fig. 5E,G). However, the time of the shift in preference between UV and the alternative stimulus was delayed after exposure to UV (from ~12 s to ~17 s), and advanced after exposure to blue or green (to ~10 s; Fig. 5F,H).

### Classical aversive conditioning further decreases the attractiveness of trained stimuli

**Figure 6:**
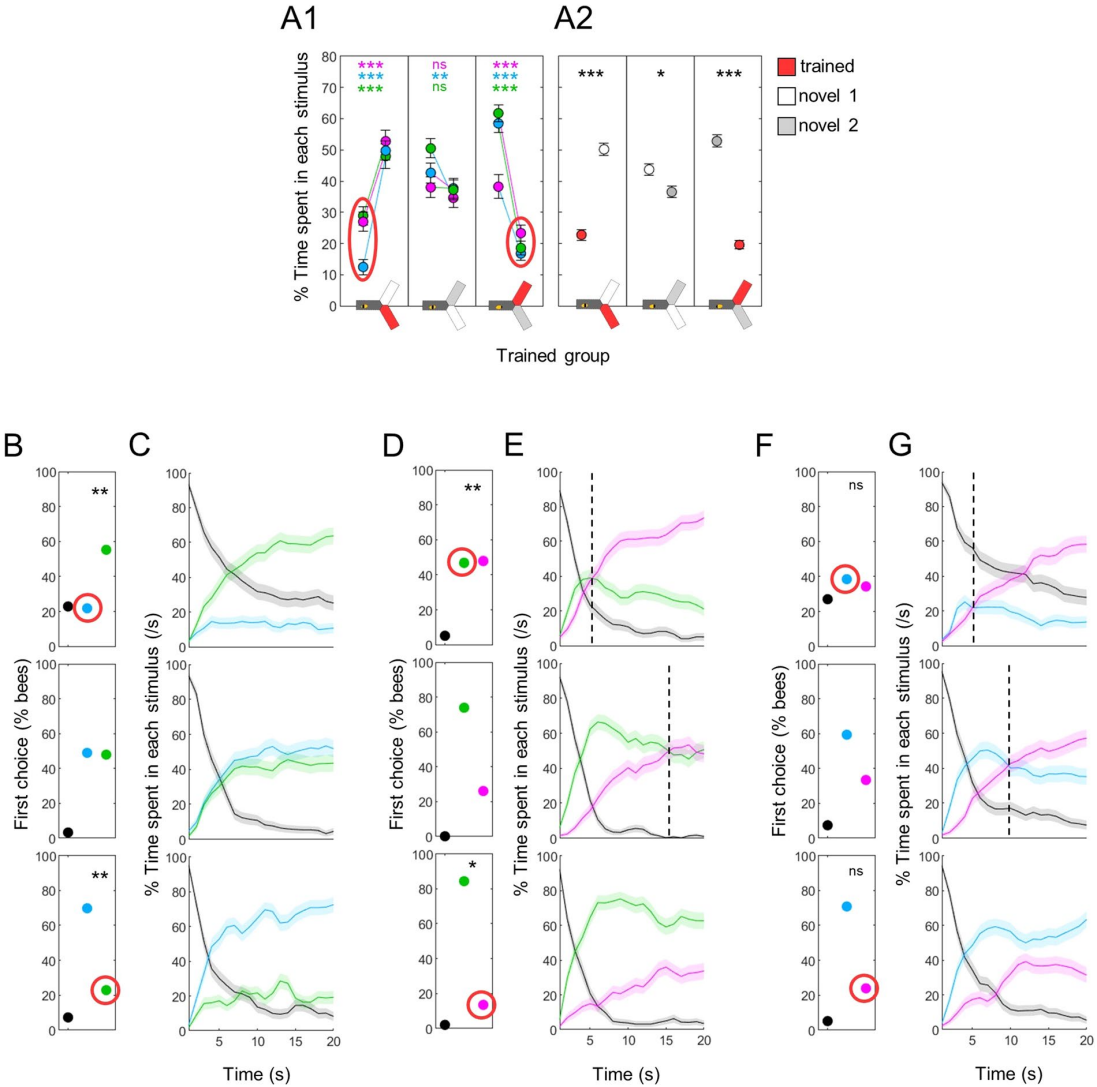
Phototaxis is further modified by training. (**A**) Percentage of time spent in each stimulus. A1 presents the data for each individual group (UV-, blue- and green-trained bees), A2 presents the pooled data. Bees avoided the wavelength that had been paired with shocks. Wilcoxon signed rank tests with FDR correction. (**B**) First choice in blue versus green tests was modified after training (trained wavelength circled in red). χ^2^ tests against bees trained to UV (middle row). (**C**) Time course of blue vs green test. The bias in preference was maintained throughout the test. (**D**) First choice for UV versus green was modified by training. χ^2^ tests against bees trained to blue (middle row). (**E**) Time course of UV vs green test. The time at which the switch in preference occurred (dotted lines) was modified when one of the test wavelength had been paired with shocks. (**F**) First choice for UV versus blue was not significantly modified by training. χ^2^ tests against bees trained to green (middle row). (**G**) The time at which the switch in preference occurred (dotted lines) was modified after one of the test wavelength had been paired with shocks. n = 288, *p < 0.05, **p < 0.01, ***p < 0.001.

**Figure 7:**
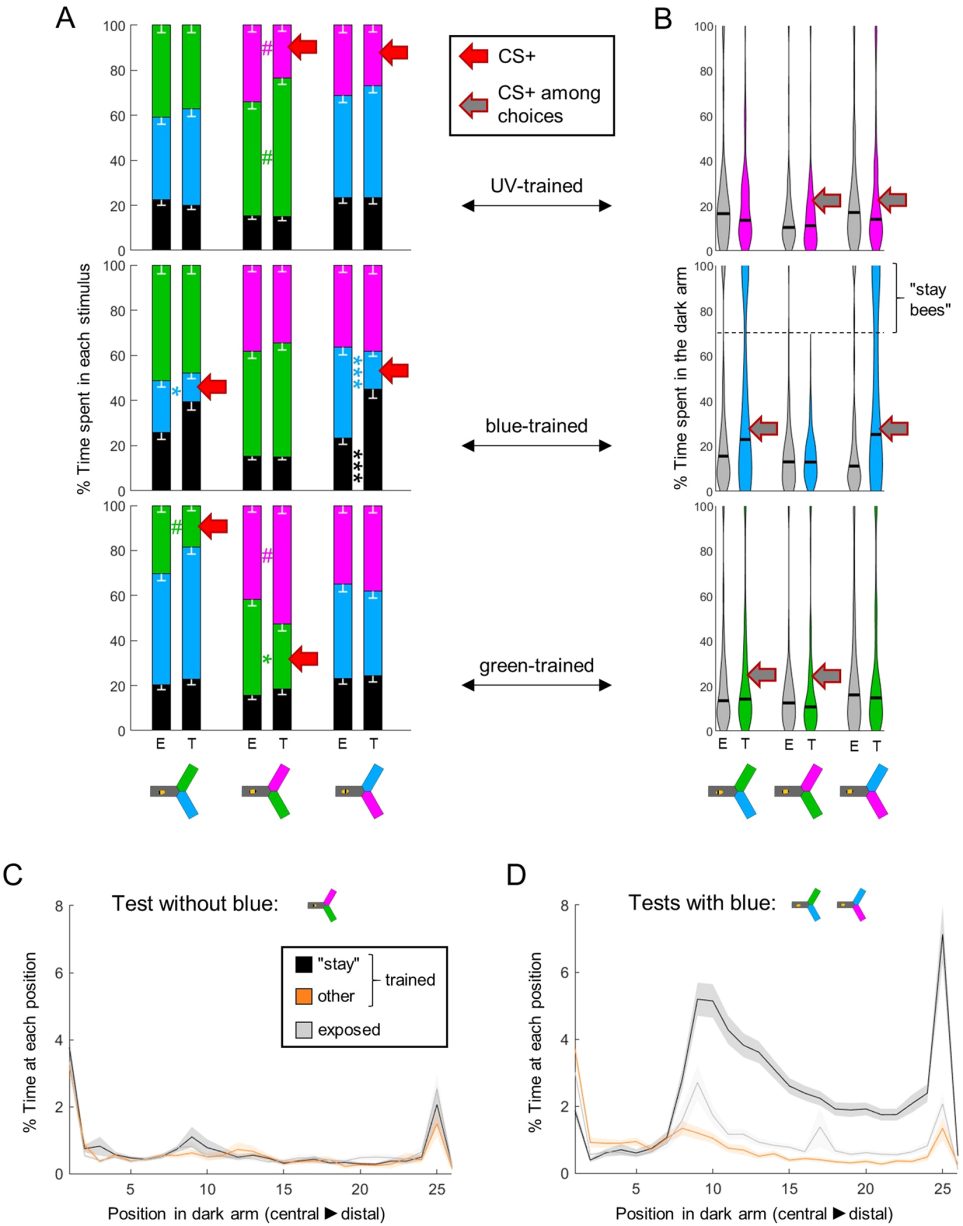
Learning strategy depends on the trained wavelength. (**A**) During training, shocks were paired with one wavelength (CS+, red arrow) in trained animals (T), while exposed bees (E) received unpaired shocks. For each group of bees, the percentages of time spent in the environments available during a given test (e.g. dark, blue and green) were plotted and stacked into a single bar. Since we included all environments, the resulting stack always totalize 100% (the whole test duration). Data is represented as mean – s.e.m.; Mann-Whitney U tests comparing E to T, corrected with FDR. (**B**) Distribution of the percentage of time spent in the dark arm. After training with blue, many bees remained in the dark arm rather than making a choice if blue was present. We categorized those bees that stayed in the dark >70% of the time during at least one test as “stay bees”. (**C**) Spatial density of bees within the dark arm, during the test without blue (n_stay_ = 53; n_other_ = 43; n_exposed_ = 96). (**D**) Same as C during tests including blue. A clear threshold is visible around position 9 that “stay bees” do not cross. n = 576, *p < 0.05, **p < 0.01, ***p < 0.001, # 0.05 > p > corrected threshold.

While exposure was sufficient to create a mild aversion to a wavelength, we expected the aversive training to trigger a more pronounced avoidance. Indeed, irrespective of the wavelength used, bees spent very little time in a trained stimulus, preferring the novel ones (Fig. 6A1-2; Wilcoxon signed rank tests corrected with FDR for pooled data, trained vs novel1: z = −7.412, p < 0.001, novel1 vs novel2: z = 1.580, p = 0.042, trained vs novel2: z = 9.749, p < 0.001). This aversion for the trained light could be seen from the very beginning of the test in all cases: when compared to bees for which both wavelengths were novel (i.e. bees trained to the 3^rd^ wavelength), a shift in the first choice could be measured in blue vs green tests (Fig. 6B, χ^2^ tests, blue trained: χ^2^(1,192) = 8.38, p = 0.004, green trained: χ^2^(1,192) = 11.90, p < 0.001) and in tests opposing green to UV (Fig. 6D, χ^2^ tests, green trained: χ^2^(1,288) = 11.91, p < 0.001, UV trained: χ^2^(1,288) = 4.43, p = 0.035). Blue vs UV tests followed a similar but non-significant trend (Fig. 6F, χ^2^ tests, blue trained: χ^2^(1,288) = 2.03, p = 0.154, UV trained: χ^2^(1,288) = 2.42, p = 0.120). As was already the case after exposure, this bias in preference was maintained throughout the test in the blue vs green configuration (Fig. 6C). For tests including UV, the major effect was again in the dynamics: the shift in preference occurred only ~5 s after the start of the test when the bees were trained against UV (whereas it took 10–15 s for control bees to reach this tipping point), and was delayed beyond the 20 s of the test after training against blue or green (Fig. 6E,G). Direct comparisons between the trained and unpaired groups confirmed that classical aversive conditioning further reduced the time spent in the trained wavelength during differential tests (Fig. 7A, Suppl. Table 1).

### The avoidance strategy depends on the trained wavelength

In differential tests, bees had 2 possibilities to avoid the trained wavelength: they could choose the arm with the alternative wavelength, or they could remain in the dark arm (where they always started). Which of these options did they use? We compared the behaviour of trained bees to the behaviour of exposed (unpaired) bees to answer this question, and found that once more, their strategy was dependent on the wavelength. Training against UV or green only shifted the preference of the bees to the alternative wavelength available, whereas training against blue did not affect the time spent in the alternative wavelength (Fig. 7A, Suppl. Table 1; note that the small effect sizes are due to exposed bees already exhibiting some aversion towards the light as was shown in Fig. 5). Instead, training against blue increased the time spent in the dark (Fig. 7A, Suppl. Table 1, also visible in the upper panels of Fig. 6C–G). We confirmed this observation by looking more closely at the time spent by individual bees inside the dark arm (Fig. 7B). Strikingly, some of the bees trained to associate blue with electric shocks spent most of their time in the dark, specifically in tests in which blue was presented. This specificity is important, because it means that positive phototaxis was not generally decreased in those bees.

To better understand this behaviour, we separated bees that stayed in the dark arm over 70% of their time during at least one test (any test), termed “stay bees” (n = 53), from the remainder bees (n = 43). In tests without blue, these two subgroups behaved similarly inside the dark arm (Fig. 7C). When the blue light was present, “stay bees” not only remained in the dark arm, they remained in the far end of that arm. A clear threshold was visible around position 9, which corresponds to a distance of ~ 5 cm from the decision point (Fig. 7D). At this distance, the blue light had a viewing angle of ~12.5° horizontally and ~15.9° vertically. It is interesting that this is within the range of the viewing angle necessary for bees to evaluate chromaticity (~15°)^14^. However, we have to exert caution as this also corresponds to the position of a slit through which air is extracted from the yAPIS, and we noted earlier that bees tend to turn at this position even in the absence of any light stimulus^23^.

### The trained aversion is superimposed on intensity-driven preferences

In all the results presented so far, light intensities were calibrated to ensure equal preference between any 2 wavelengths in naïve bees (Fig. 1E). In this final experiment, we investigated how deviations from these settings would affect the bees’ performance. Would the intensity difference completely override learning, such that bees would always go to the brightest light? Or, in other words, is there a hierarchy in how bees integrate information about a light? We addressed this question by training bees with either blue or green, and then by varying the intensity of one stimulus during the tests (either the trained one or the novel one; see Fig. 2B for protocol). Hence we had 4 groups of trained bees: “blue trained & blue varied”, “blue trained & green varied”, “green trained & green varied” and “green trained & blue varied”. We only used blue and green lights in this experiment because the bees’ behaviour was easier to interpret in these differential tests. We also included 2 groups of control (naïve) bees which did not receive any stimulation during the training phase (to avoid the exposure effect demonstrated previously): “blue varied” and “green varied”. Each of these control groups was randomly split in 2 to replicate the 4 groups of trained bees (i.e. green was considered the trained wavelength for half of the bees, and blue for the other half). The aim of this slightly complicated design was twofold: 1) cancelling wavelength-specific effects by having symetrical roles for blue and green; and 2) checking if the bees’ behaviour was dictated by the difference in intensities independently of the role of the varied stimulus (trained or novel). The behavioural pattern that we describe in detail below was the same in all groups (Suppl. Fig. 2): neither which wavelength was trained nor which stimulus was varied in the test changed the results. Therefore, we pooled the data, with the tests classified into 3 categories: “both lights calibrated”, “trained dimmer (than novel)” and “trained brighter (than novel)”.

**Figure 8:**
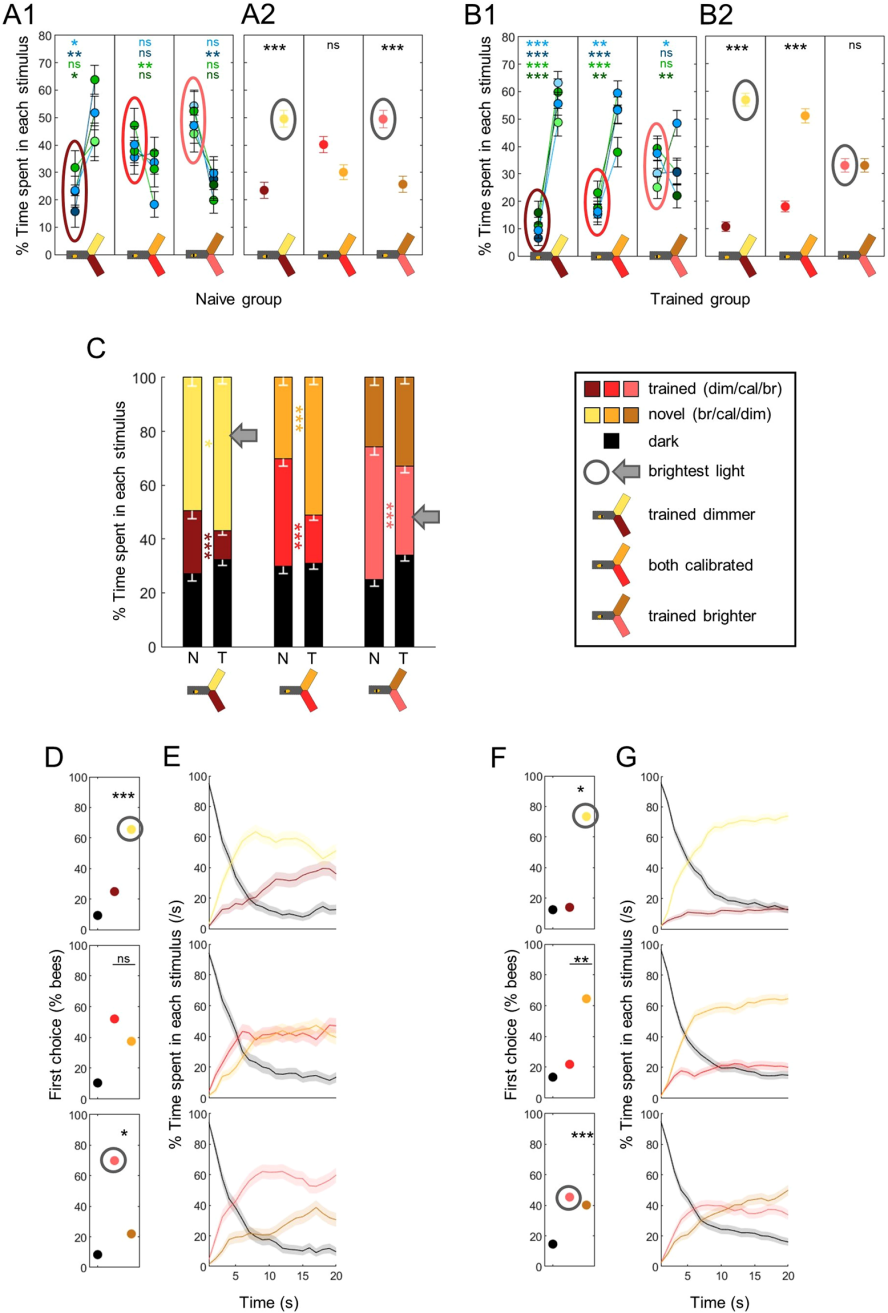
The trained aversion is superimposed on the innate preference. During training, shocks were paired with one wavelength for trained animals (T), while naïve bees (N) were not stimulated. Lighter colors indicate lights brighter than the calibrated reference, darker colors indicate lights dimmer that the calibrated reference. (**A**) Percentage of time spent in each stimulus, for naïve bees. A1 presents the data for each pair of trained-varied wavelength (blue-green stats are in blue, blue-blue in dark blue, green-blue in green and green-green in dark green), A2 presents the pooled data. Wilcoxon signed rank tests with FDR correction. (**B**) Same as in A but for trained bees. (**C**) Percentages of time spent in the environments available during a given test (dark, trained and novel) plotted and stacked into a single bar. Trained bees spent less time than naive bees in the light previously associated with shocks, independently of its relative brightness. Mean – s.e.m, Mann-Whitney U tests comparing N to T, corrected with FDR. (**D**) The first choice of naive bees was always biased toward the brightest light (circled in grey). χ^2^ tests against calibrated intensities (top row), or against a random choice for this reference test. (**E**) Time courses for naïve bees. The preference was maintained throughout the test. (**F**) The first choice of trained bees was modulated by both training and brightness. χ^2^ tests against calibrated intensities (top row), or against a random choice for this reference test. (**G**) Time courses for trained bees. The first choice was corrected during the test if it contradicted the training paradigm. n = 576, *p < 0.05, **p < 0.01, ***p < 0.001, ns: p > 0.05.

As expected, deviations from the calibrated settings induced biases in preference in naïve bees, such that they always chose the brightest of the two wavelengths (Fig. 8A1–2; Wilcoxon signed rank tests corrected with FDR for pooled data, trained dimmer: z = −2.551, p < 0.001, both calibrated: z = 1.909, p = 0.056, trained brighter: z = 2.264, p < 0.001). This preference for the brightest light was also obvious in the bees’ first choices (Fig. 8D; χ^2^ tests against calibrated lights, trained dimmer: χ^2^(1,96) = 16.49, p < 0.001, trained brighter: χ^2^(1,96) = 6.39, p = 0.011), and was maintained throughout the tests (Fig. 8E). Trained bees, on the other hand, only showed a preference for the brightest light when it was novel (Fig. 8B; Wilcoxon signed rank tests corrected with FDR, trained dimmer: z = −8.868, p < 0.001, both calibrated: z = −7.120, p < 0.001, trained brighter: z = 0, p = 1). Direct comparisons confirmed that trained bees spent less time than naïve bees in the wavelength associated with shocks in all tests, independently of relative intensities (Fig. 8C; Suppl. Table 1). Thus, the intensity bias did not override the learned aversion. But the aversive training by itself also did not fully account for the bees’ behaviour: the shifts in intensity modulated both the time spent in each light (Fig. 8B) and first choice (Fig. 8F; χ^2^ tests against calibrated lights, trained dimmer: χ^2^(1,192) = 4.34, p = 0.037, trained brighter: χ^2^(1,192) = 26.68, p < 0.001). In particular, when the trained wavelength was brighter, trained bees still had a slight preference for it at the beginning of the test before switching to the novel (but dimmer) light (Fig. 8G). Taken together, these results demonstrate that bees clearly integrated both intensity and experience into an overall valence when comparing the two stimuli.

## Discussion

In this study, we explored how previous experience through exposure and aversive training shaped honey bees’ response to light, while controlling the possible confounding effects of light properties (wavelength and relative intensity). Our data suggest that phototaxis is a highly dynamical response and sensitive to all the factors listed above, which are integrated into an overall valence for a specific light. Exposure had similar effects to a mild aversive training, which is maybe best understood by considering that the bees likely go towards the lights because they want to escape the apparatus. Since the light they have seen before has already proven disappointing (they found no escape), the new light may appear more promising by comparison. Alternatively, they may have a general bias towards new stimuli, as has been reported before^25^.

In our system, naïve bees moved towards any of the 3 wavelengths in absolute tests. However, their reaction time and their persistence depended on the wavelength considered, which was the first indication of wavelength-specificity. This was confirmed when evaluating the behavioural plasticity of this circuit: both reaction time and persistence were independently and selectively modified after training against some wavelengths but not others. Absolute phototaxis towards UV seemed to be immune to aversive training, while the same behaviour towards blue was especially labile. Similar selective plasticity in the blue range was obtained by other studies using a comparable approach^26,27^. This could be linked to the relative efficiency of each wavelength in eliciting positive phototaxis, which was evident in our calibration data (Fig. 1B; the absolute photon counts required to elicit equal preference scaled 1:63:29 for UV:blue:green) and had been established before^18,28^. In addition, this is not the first time that wavelength-specific learning effects have been observed in honey bees. For example, learning speed (how many trials are necessary) is fastest around 420 nm and slowest around 494 nm during appetitive conditioning^29^.

Importantly, wavelength-specificity does not imply true colour vision^9^, and it remains unclear whether our training paradigm relied on true colour vision or on photoreceptor-specific channels. Neurons dowstream of photoreceptors can be classified into three groups based on their chromatic sensitivity^30–32^. Broad-band neurons respond indifferently to all wavelengths, whereas narrow-band neurons only respond to a limited range of wavelengths. Finally, colour-opponent neurons are excited by some wavelengths but inhibited by others^33,34^, thus creating an intensity invariant read-out of the light spectrum supporting true colour vision^9^. Individual photoreceptors hence contribute to multiple streams of information^30,35–38^. A wavelength-specific behaviour may be mediated by a specialized circuit with a dedicated photoreceptor that does not contribute at all to true colour vision^39^. As a consequence, at least two hypothetical circuits could explain our current results, and further experiments will be needed to choose between them. In the simpler scenario, plastic synapses would be located directly between photoreceptor cells and the putative pooling neurons mediating phototaxis^17^. In this view, the valence of a given light would be directly related to the activity of the pooling neurons, and independent of true colour vision. In appetitive contexts, however, visual learning does rely on colour-opponent neurons^40,41^. In addition, several studies suggest that different properties of a visual stimulus are processed independently in the insect brain: colour, motion and timing of a stimulus are segregated in the lobula of a bumble bee^36,37^, and colour and brightness information rely on distinct populations of neurons in the fruit fly^42,43^. This suggests an alternative scenario, in which these multiple pathways would converge into a common integrator in order to attribute an overall valence to the stimulus. Although slightly more complex, such circuitry has been reported before^42,44^. If true, this integration is likely to happen centrally, in regions such as the anterior optic tubercles, the mushroom bodies or the central complex, which have been implicated in colour learning in a variety of insects^45–47^. In any case, it is interesting to note that our bees never generalized the training paradigm by avoiding all lights, suggesting that phototactic learning is intrisically wavelength-specific.

The temporal complexity that we observed may be explained by assuming at least 3 steps in the decision-making process: an approach phase, a first choice and a continuous re-assessment of the decision. A puzzling element of our results is that, even though direct aversive training of the green or UV lights could not abolish absolute phototaxis towards these lights, the adjunction of the trained blue light in another arm could. Postulating the existence of an all-or-nothing initial step, whereby bees decide whether to approach the lights or not, would resolve this paradigm. After the bees approached the lights, in differential tests they then had to choose which light stimulus to enter. We found that this first choice depended on the wavelengths and relative intensities of the two lights, and could be modified by training. Finally, we observed shifts in preference during the 20 s of the tests. In particular, naïve bees initially chose blue or green against UV, but by the end of the test this preference was reversed. Failure to find an exit within the first stimulus, or physiological adaptation to the light, might have slowly decreased the attractiveness of this stimulus until it eventually became less attractive than the alternative, originally unfavoured option. In natural settings, this constant re-evaluation is likely important to allow bees to search multiple options in a hierarchical order, instead of getting stuck in a dead-end. Consistent with this hypothesis, we observed that previous experience decreasing the initial valence of a light could shift the time at which the equilibrium between the two lights was reached (Figs 5F–H and 6E–G).

The dynamics of blue/green vs UV tests may thus be explained by a combination of 3 factors: the strong and resilient phototactic drive elicited by UV, the slow devaluation of explored stimuli and our calibration protocol (which took equal time spent as criterion). It is likely that our settings created an initial imbalance, i.e. that UV was dimmer from the bees’ perspective, which was required for bees to visit the alternative wavelength for some time. From our results, we expect that calibrating the first choice instead would result in a longer time spent in the UV, since its decay in valence is slower than for blue or green. Another possibility would be that UV is integrated differently from the other wavelengths and that this impacts how it is compared. Recent data supports this hypothesis: UV induces configural processing of olfactory-visual compounds (i.e. the compound is different from the sum of its components), whereas blue and green produced elemental compounds^48^. A different comparison mechanism could explain that exposure to blue/green did not impact the bees’ behaviour during blue/green vs UV tests, but did so during blue vs green tests (Fig. 5E–H).

Overall, our results demonstrate that animals with a complex visual system, like the honey bee, can exhibit refined phototaxis behaviour. In particular, they can adapt this behaviour based on individual experience. These results also open new questions that should be addressed in future studies. We are especially curious about the ecological relevance of such modulation, as well as the mechanisms supporting it.

## Methods

### Honey bees

Bees were taken from colonies housed on the roof of the University of Konstanz, Germany. The bees participating in the experiments were caught as they left the colony, and were thus most likely foragers. They were introduced inside the behavioural training and testing chamber yAPIS immediately after being caught.

To check calibrated intensities, 3 groups (one for each wavelength pair) of 18 bees were tested (total n = 54). Conditioning experiments were done with 6 treatment groups in parallel (3 paired and 3 unpaired) of 96 bees each, total n = 576. The 3 CS-only groups contained 48 bees each (total n = 144). The experiment in which green was trained with 12 pairing trials included 2 groups (paired + unpaired) of 48 bees each (total n = 96). Finally, the experiment in which light intensities were varied included 6 groups ((2 symmetrical pairing and 1 naïve control) × 2 wavelengths varying in intensity) of 48 bees each (total n = 288).

### Training and testing apparatus: yAPIS

Honey bees were trained in an automated y-maze consisting in 3 arms of equal length (14 cm) at 120° from each other: yAPIS (Fig. 1A). This apparatus has been described in detail before^23^. Briefly, the bee was tracked in real time within the y-maze so that lights placed underneath the transparent floor could be switched on relative to its position. We used three types of LEDs, spanning different wavelengths: human green (λ = 520 nm), human blue (λ = 465 nm) and ultraviolet (λ = 375 nm; Fig. 1A,B), that we refer to as green, blue and UV for simplicity. Bee photoreceptors differ in quantum efficiency, with S-photoreceptors being most sensitive, and L-photoreceptor least (Fig. 1B)^49^. We calibrated light intensities behaviourally, so that bees had an equal preference in a two-choice test (Fig. 1E, Wilcoxon signed rank tests, blue vs green: z = −1.154, p = 0.248, green vs UV: z = −0.327, p = 0.744, blue vs UV: z = 0.544, p = 0.586). This corresponded to intensity settings of 64%, 44% and 2,4% of the maximum intensity, corresponding to a total photon counts of 898.10^12^, 1964.10^12^ and 31.10^12^ quanta/cm^2^/s, for green, blue and UV respectively. The relative excitation of each photoreceptor was calculated by multiplying each stimulus spectrum by the response function of the photoreceptor and integrating the area under the resulting curve. It should be noted that these calculations relate to single photoreceptor cells. The total sensitivity of the eye is also influenced by other factors, such as the relative density of photoreceptor cells. It also does not account for further processing inside the optic lobe. These calibrated intensity settings were kept for all experiments except the one in which light intensities were varied for green or blue. In this case, we also used dimmer lights: 32% for green, 12% for blue; and brighter lights: 96% for green, 76% for blue.

### Training procedures

During training, one light was turned on throughout the apparatus, so that the bee was passively exposed, and the shocks were delivered according to group treatment. This was a classical Pavlovian conditioning paradigm. Overall, 4 different protocols were used for training (Fig. 2). (1) In **trained** animals the light was switched on in all three arms for 10 s, and the US (electric shocks) was delivered simultaneously. The US consisted of a train of mild electric shocks (2 Hz, 10 V, 200 ms, 20 shocks in total) delivered by the electric grid placed on the floor and ceiling of each arm. Four training trials with an inter-trial interval of 30 s were delivered, unless stated otherwise. (2) For **unpaired** animals, light was switched on in all three arms for 10 s, and electric shocks were delivered for 10 s in the middle of the 30 s intertrial interval. (3) In **CS-only** bees the shocks were completely removed, leaving only the lights. (4) **Naïve** animals spent the same amount of time in the apparatus but did not receive any treatment during the training phase (i.e. they stayed in the dark until the tests).

After training, animals were left in the dark yAPIS for 4 minutes, which is sufficient to reach dark adaptation in photoreceptors^17,50^. At the start of the tests the position of the bee was evaluated and lights were presented in the other two arms of the apparatus, such that the bee always started in the dark arm. Tests (20 s) were separated by 30 s intervals, and test order was balanced across bees. In the first conditioning experiment, all bees were tested both for differential and for absolute phototaxis (6 tests, Fig. 2A). In the experiments in which light intensity was varied for a wavelength (Fig. 2B), bees were tested first with 3 differential tests: “wavelength 1 calibrated vs. wavelength 2 calibrated”, “wavelength 1 calibrated vs. wavelength 2 dimmer” and “wavelength 1 calibrated vs. wavelength 2 brighter”. To check that bees were still positively phototactic towards the new stimuli used, they were then also tested with 2 absolute tests, one with “wavelength 2 dimmer” and the other with “wavelength 2 brighter” (data shown in Suppl. Fig. 1).

### Data analysis

To analyse the overall results of the first conditioning experiment, we differentiated the behaviour of the bees towards novel wavelengths, towards a wavelength they had seen but not paired with shocks (exposed) and towards a wavelength they had been trained to (Fig. 2A). Only control bees (from the unpaired groups) were included for the analysis of behaviour towards novel and exposed wavelengths. In the experiment in which light intensity was varied (Fig. 2B), the tests were pooled within 3 categories: “trained calibrated vs novel calibrated”, “trained dimmer than novel” and “trained brighter than novel”. This was done after showing that the behaviour was consistent across both trained wavelengths and irrespective of whether the wavelength varying in intensity was the trained one or the novel one (Suppl. Fig. 1).

The yAPIS system collected the following data onto a log file: time series of the position of the bee along the arm, the arm the bee was in, each electric shock, the current flow during the shock and the lights on/off events with stimulus intensity. The data was analysed using custom scripts written in Python 3.7 and Matlab R2018b. Bees that moved slower than 6 mm/s on average during the test phase were excluded, and new bees were measured instead. Direct comparisons of the two lights in choice tests were performed using Wilcoxon signed rank tests. Mann-Whitney U tests were performed to evaluate the change in distributions between a control group and the trained group. To compare the proportions of bees choosing first one wavelength or the other (first choice evaluation), Chi-square tests were performed either against the control group or against chance level. Analysis of the delay before entering a wavelength used a one-way ANOVA followed by a post-hoc Tukey’s Honest Significant Difference (HSD) test. When appropriate, *p* values were corrected for multiple comparisons using a False Discovery Rate (FDR) procedure, which controls for both type I and type II errors^51^.

## Supplementary information

Supplementary information.
