# Supplementary material for: Complexity and plasticity in honey bee phototactic behaviour
Source: Sci Rep. 2020 May 12;10:7872. doi: 10.1038/s41598-020-64782-y (PMC7217928; doi:10.1038/s41598-020-64782-y)
Supplement: Supplementary file 1 — Supplementary information. [file 41598_2020_64782_MOESM1_ESM.pdf]

Complexity and plasticity in honey bee phototactic behaviour

Morgane Nouvian[1,*] , C. Giovanni Galizia[1,2]

[1]Department of Biology, University of Konstanz, Konstanz, Germany

[2]Centre for the Advanced Study of Collective Behaviour, University of Konstanz, 78464, Konstanz, Germany

* Correspondence: morgane.nouvian@uni-konstanz.de

Supplementary Information

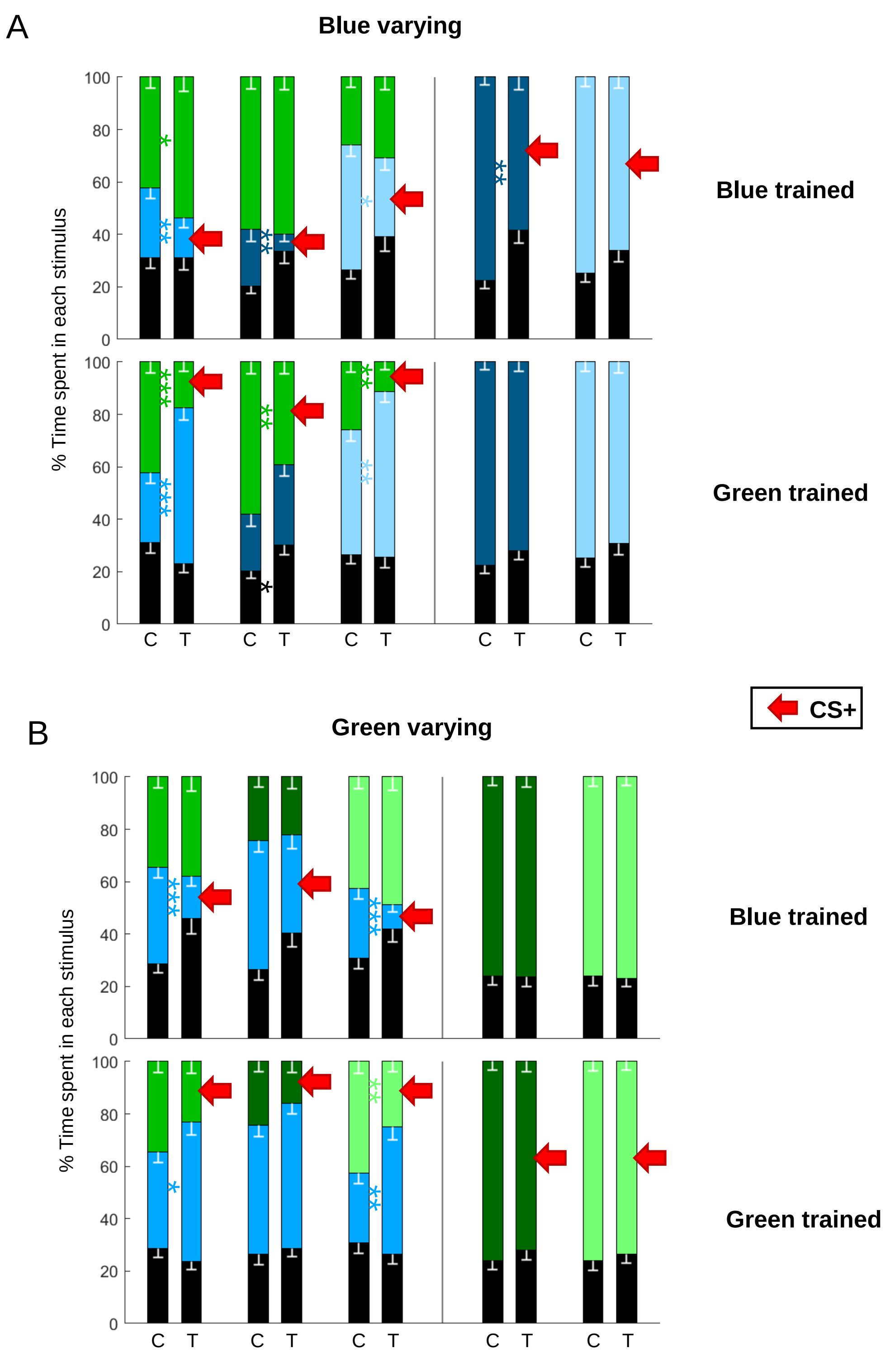

<u>Supplementary Fig. 1</u>: Neither the identity of the CS+ (blue or green) nor which stimulus (CS+ or novel) varied in intensity affected the interaction between learning and intensity-driven preference. A. Blue light varied. B. Green light varied. Lighter colors indicate lights brighter than the calibrated reference, darker colors indicate lights dimmer that the calibrated reference. n=48 bees x 6 groups, Mann-Whitney U tests comparing C to T, corrected with FDR, * $p<0.05$, ** $p<0.01$, *** $p<0.001$.

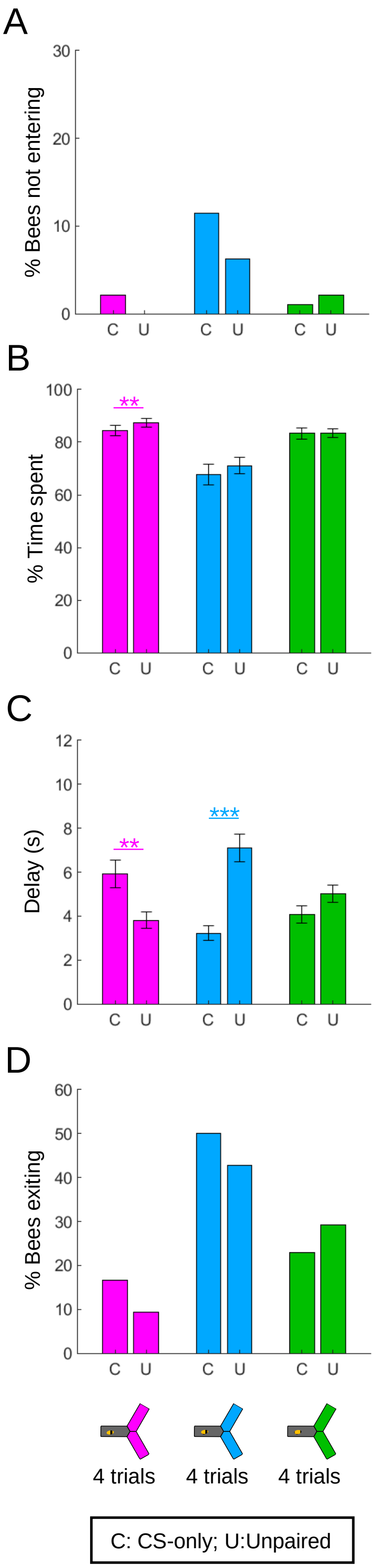

<u>Supplementary Fig. 2</u>: Comparison of unpaired and CS-only groups during absolute tests. A. Percentage of bees not entering the light. χ2 tests. B. Percentage of time spent in the light. Wilcoxon signed rank tests. C. Delay before entering the light. Anova. D. Percentage of bees exiting the light before the end of the test. χ2 tests. ** p<0.01, *** p<0.001.

Group	Test	Colour	$n_t = n_c$	z	p	pFDR
B	B vs G	D	96	1.7495	0.0802	0.1804
		B	96	-2.7741	**0.0055 ****	**0.0165 ***
		G	96	-0.425	0.6708	0.7546
	G vs U	D	96	0.7234	0.4694	0.6035
		G	96	0.9442	0.3451	0.6212
		U	96	-0.7657	0.4438	0.6657
	B vs U	D	96	3.9747	**<0.0001 *****	**<0.0006 *****
		B	96	-5.1045	**<0.0001 *****	**<0.0006 *****
		U	96	0.2283	0.8194	0.8194
G	B vs G	D	96	0.6364	0.5245	0.7868
		B	96	1.8946	0.0581	0.1308
		G	96	-2.3888	**0.0169 ***	0.0507
	G vs U	D	96	-0.2104	0.8334	0.9375
		G	96	-3.2670	**0.0011 ****	**0.0099 ****
		U	96	2.4770	**0.0132 ***	0.0594 #
	B vs U	D	96	0.1520	0.8792	0.8792
		B	96	-0.9356	0.3495	0.6291
		U	96	0.6216	0.5342	0.6868
U	B vs G	D	96	-0.3286	0.7425	0.8353
		B	96	1.2821	0.1998	0.5995
		G	96	-0.8085	0.4188	0.7539
	G vs U	D	96	-0.5299	0.5962	0.7665
		G	96	2.6772	**0.0074 ****	0.0666 #
		U	96	-2.3823	**0.0172 ***	0.0774 #
	B vs U	D	96	-0.3091	0.7572	0.7572
		B	96	1.0634	0.2876	0.6471
		U	96	-0.7206	0.4712	0.7068
Varying intensities	trained dimmer	D	96	-1.2515	0.2108	0.2710
		CS+	96	4.5194	**<0.0001 *****	**<0.0001 *****
		CS-	96	-2.2480	**0.0246 ***	**0.0442 ***
	both calibrated	D	96	0.9537	0.3402	0.3402
		CS+	96	6.3524	**<0.0001 *****	**<0.0001 *****
		CS-	96	-4.8669	**<0.0001 *****	**<0.0001 *****
	trained brighter	D	96	-1.5581	0.1192	0.1788
		CS+	96	3.9228	**0.0001 *****	**0.0002 *****
		CS-	96	-1.2138	0.2248	0.2529

Supplementary Table 1: Summary of statistical results for Fig. 7A and 8C. Wilcoxon signed-rank tests, "p FDR" indicates the p value corrected with FDR, * p<0.05, ** p<0.01, *** p<0.001, # 0.05>p>corrected threshold.
